# Fungal endophytes of *Catharanthus roseus* enhance vindoline content by modulating structural and regulatory genes related to terpenoid indole alkaloid biosynthesis

**DOI:** 10.1038/srep26583

**Published:** 2016-05-25

**Authors:** Shiv S. Pandey, Sucheta Singh, C. S. Vivek Babu, Karuna Shanker, N. K. Srivastava, Ashutosh K. Shukla, Alok Kalra

**Affiliations:** 1 Microbial Technology Department, CSIR-Central Institute of Medicinal and Aromatic Plants, Lucknow-226015, India; 2 CSIR-Central Institute of Medicinal and Aromatic Plants, Research Centre, Allalasandra, GKVK Post, Bangalore-560065, India; 3 Analytical Chemistry Department, CSIR-Central Institute of Medicinal and Aromatic Plants, Lucknow-226015, India; 4 Plant Physiology Department, CSIR-Central Institute of Medicinal and Aromatic Plants, Lucknow-226015, India; 5 Biotechnology Division, CSIR-Central Institute of Medicinal and Aromatic Plants, Lucknow-226015, India

## Abstract

Not much is known about the mechanism of endophyte-mediated induction of secondary metabolite production in *Catharanthus roseus*. In the present study two fungal endophytes, *Curvularia* sp. CATDLF5 and *Choanephora infundibulifera* CATDLF6 were isolated from the leaves of the plant that were found to enhance vindoline content by 229–403%. The isolated endophytes did not affect the primary metabolism of the plant as the maximum quantum efficiency of PSII, net CO_2_ assimilation, plant biomass and starch content of endophyte-inoculated plants was similar to endophyte-free control plants. Expression of terpenoid indole alkaloid (TIA) pathway genes, geraniol 10-hydroxylase (*G10H*), tryptophan decarboxylase (*TDC*), strictosidine synthase (*STR*), 16-hydoxytabersonine-*O*-methyltransferase (*16OMT*), desacetoxyvindoline-4-hydroxylase (*D4H*), deacetylvindoline-4-*O*-acetyltransferase (*DAT*) were upregulated in endophyte-inoculated plants. Endophyte inoculation upregulated the expression of the gene for transcriptional activator octadecanoid-responsive Catharanthus AP2-domain protein (*ORCA3*) and downregulated the expression of Cys2/His2-type zinc finger protein family transcriptional repressors (*ZCTs*). The gene for the vacuolar class III peroxidase (*PRX1*), responsible for coupling vindoline and catharanthine, was upregulated in endophyte-inoculated plants. These endophytes may enhance vindoline production by modulating the expression of key structural and regulatory genes of vindoline biosynthesis without affecting the primary metabolism of the host plant.

Endophytes are microbes that are found to be present in almost all plants studied^1^. They reside inside the host plant without causing any disease symptoms or harm^2^,^3^. Most of the endophytes enter the plant through root hairs or the stomata in leaves and then disseminate systemically throughout the plant. Endophytes may be present intercellularly or intracellularly and colonize roots, aerial parts, conduction vessels and seeds^4^. Endophytes may promote host plant growth, improve nutrient supply and protect plants from both biotic and abiotic stresses^5^,^6^,^7^,^8^. Endophytic microbes are the potential source of therapeutically important bioactive natural products^9^. Few endophytic fungi produce secondary metabolites similar to their host plant e.g. vinblastine and vincristine^10^, taxol^11^, azadirachtin^12^, podophyllotoxin^13^, deoxypodophyllotoxin^14^, camptothecin^15^ and hypericin^16^. A major limitation in bioactive secondary metabolite production in present fermentation practices through the use of fungal endophytes is the instability of the expression of genes involved in the biosynthesis of the desired metabolite(s). During repeated subculturing under axenic monoculture conditions, production of the secondary metabolite may reduce substantially^17^. Therefore, the use of endophytes to enhance secondary metabolite production *in planta* could be a better approach compared to their independent culture *in vitro*.

There are many reports of endophyte-induced biosynthesis of secondary metabolites in host plants, but limited information is available about the mechanism involved. An endophytic actinobacterium *Pseudonocardia* strain YIM 63111 isolated from *Artemisia annua* induced artemisinin production in *Artemisia* plant by upregulating the expression of cytochrome P450 monooxygenase and cytochrome P450 oxidoreductase genes involved in artemisinin biosynthesis^18^. Elicitors from the endophytic fungus *Trichoderma atroviride* stimulate biosynthesis of tanshinone in the host plant by increasing the expression of genes related to tanshinone biosynthesis^19^. *Catharanthus roseus* is one of the most studied medicinal plants and is used as a model species for the study of plant secondary metabolism and plant-microbe interactions^20^,^21^,^22^,^23^,^24^. It is the sole source of antitumor bisindole alkaloids [belonging to the class terpenoids indole alkaloids (TIAs)] vinblastine and vincristine, which are extensively used in cancer chemotherapy. Due to a very low production of vinblastine and vincristine *in planta* and their large demand, they are exorbitantly priced. This great commercial importance has led to major efforts to increase the *in planta* content of these metabolites. Vinblastine and vincristine are produced by the condensation of monomeric TIAs, vindoline, and catharanthine. Due to complicated structures of these alkaloids, their chemical synthesis in large scale is not economically feasible^25^,^26^. Diverse approaches such as transgenic generation, tissue culture practices, phytohormone treatments are being attempted to achieve enhanced production of important TIAs. Most of the genes that encode enzymes for TIA biosynthesis and regulatory components such as transcriptional activators and repressors have been identified^20^,^27^,^28^,^29^,^30^,^31^ (Fig. 1). Transgenic and tissue culture approaches have their own limitations. It is difficult to increase the *in planta* content of bisindole alkaloids due to their cytotoxicity; a better approach may be to produce monomers and semisynthetically fuse them to produce bisindoles^20^. Use of endophytes to enhance secondary metabolite production in the host plant could be a sustainable approach. Earlier, we have shown that bacterial endophytes enhance the *in planta* content of key TIAs as well as plant growth and biomass^32^. In the present study, efforts were made to identify and characterize fungal endophytes capable of enhancing the vindoline content in *C. roseus* and to study the possible mechanism involved. Towards this end, fungal endophytes were isolated from alkaloid-rich genotype (cv. Dhawal) of *C. roseus*^20^ and their effect was checked on another *C. roseus* genotype (cv. Prabal) that produces a comparatively lower amount of TIAs. The idea was to explore the role, if any, of endophytes isolated from an alkaloid-rich genotype in improving the alkaloid content of low-alkaloid genotypes.

## Results

### Isolation of fungal endophytes that enhance the vindoline biosynthetic potential of *C. roseus*

**Figure 2:**
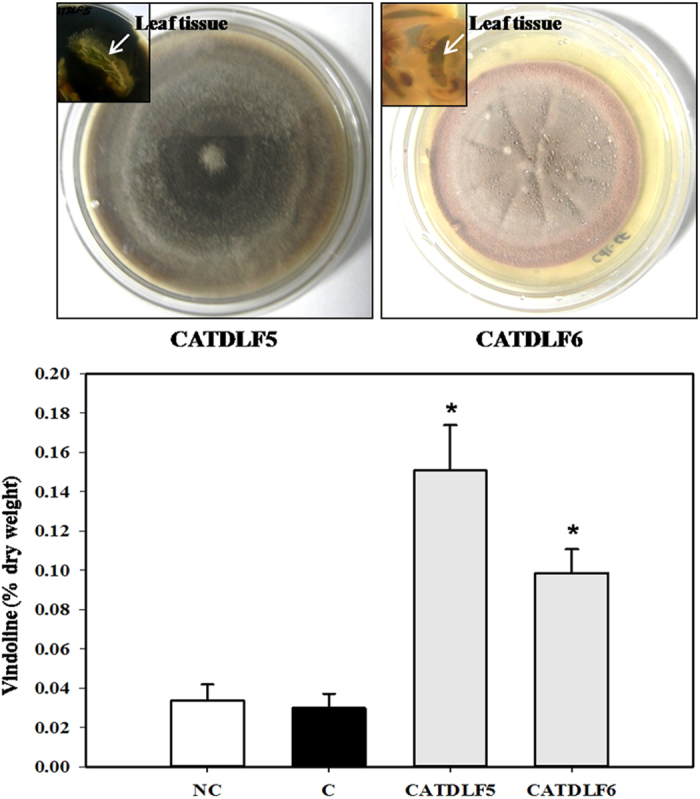
Fungal endophytes isolated from *Catharanthus roseus* and their effect on vindoline content in plant leaves. Upper panel: Fungal endophytes isolated from the surface sterilized leaves of *C. roseus* (cv. Dhawal) plants. Surface sterilized leaves were cut into small pieces and kept on potato dextrose agar plate, whereby endophytes were found to originate from the margin of the pieces (inset picture). Lower panel: Endophyte-free *C. roseus* (cv. Prabal) plants (generated from seeds treated with bactericide and fungicide) were used to study the effect of treatment with endophytes (CATDLF5 and CATDLF6 isolated from cv. Dhawal) on leaf vindoline content. Two types of controls were included in the study-(i) the endophyte-free control [C] plants that originated from *C. roseus* seeds treated with bactericides and fungicides and were thus devoid of any naturally occurring endophyte and (ii) the natural control [NC] plants that originated from *C. roseus* seeds that were not treated with any bacteriocide and fungicide and contained all the naturally occurring endophytes present in the plants. Fungal endophyte inoculums (1 × 10^8^ spore/conidia mL^−1^) prepared in phosphate buffer saline (PBS) were used to treat roots of experimental plants. The roots of both the controls–endophyte-free (C) and natural (NC) plants were treated with PBS. Third leaves of 90 d-old plants were sampled for vindoline content (% dry weight basis). As biological replicates, three plants per treatment were analyzed. For each biological replicate, three technical replicates were run on the HPLC and the mean of the three technical replicates represented the particular biological replicate. Statistical analysis was carried out for the data obtained for the three biological replicates (n = 3). Asterisks indicate significant differences as compared to the endophyte-free control (C) (Duncan’s multiple range test *P < 0.05).

A total of seven fungal endophytes were isolated from *C. roseus* genotype Dhawal and their potential to enhance vindoline content was examined in both genotypes Dhawal and Prabal. It was found that out of the seven fungal endophytes, CATDLF5 and CATDLF6, identified as *Curvularia* sp. and *Choanephora infundibulifera* by ITS sequencing, respectively (Fig. 2) were found to improve the vindoline content in genotype Prabal but could not improve the same in genotype Dhawal in the preliminary glass house trials (Supplementary Fig. S1). Therefore, to evaluate the possible mechanism involved in fungal endophyte-mediated enhancement of vindoline biosynthesis, further study was performed on genotype Prabal. The isolated endophytes were used to inoculate endophyte-free seedlings of *C. roseus* genotype Prabal. After 90 d of growth, the presence of inoculated endophytes in the leaves of endophyte-inoculated plants was examined, and vindoline content was measured. High-performance liquid chromatography (HPLC) analysis of leaves of CATDLF5- and CATDLF6-inoculated plants was performed, and vindoline content was compared with two types of controls–(i) endophyte-free control [C] and (ii) natural control [NC] plants containing the naturally present endophytes (these plants were not made endophyte-free by the treatment of fungicide and bactericide). The purpose to include the natural control was to get an idea of the effect of the natural endophytic populations occurring in the plants *vis-à-vis* the endophyte-free control plants. Leaves of CATDLF5- and CATDLF6-inoculated plants had 403% and 229% higher vindoline content as compared to that in endophyte-free control plants, respectively (Fig. 2; Supplementary Fig. S2). It was observed that endophyte-free control and natural control plants did not differ significantly in their vindoline content. To rule out the possibility of CATDLF5 and CATDLF6 endophytes producing vindoline themselves, they were examined for the presence of vindoline in their culture media (independent of host) by ethyl acetate extraction followed by HPLC. The selected endophytes were not able to produce vindoline in the culture medium (data not shown).

### Effect of CATDLF5 and CATDLF6 inoculation on photosynthetic efficiency of *C. roseus*

To examine the effect of endophytes on primary metabolism of the plant, photosynthetic efficiency of plants inoculated with CATDLF5 and CATDLF6 was measured and compared with endophyte-free control and natural control plants. Chlorophyll content, chlorophyll fluorescence, net CO_2_ assimilation, stomatal conductance and transpiration rate were measured.

Chlorophyll and carotenoid content were not affected by endophyte inoculation (Table 1). Natural control plants had ~12% higher total chlorophyll content as compared to endophyte-free control plants. Chlorophyll content in CATDLF5- and CATDLF6-treated plants were similar to endophytes-free controls. Chlorophyll fluorescence, which is believed as the signature of photosynthesis of a plant^33^ and represents functional status of photosynthetic apparatus and electron transport chain was not affected by endophyte inoculation. Fv/Fm of natural control plants was higher than that of endophyte-free control plants as well as CATDLF5- and CATDLF6-inoculated plants. The Fv/Fm indicates the quantum efficiency of photosynthesis. The Fv is the variable fluorescence, which represents Fm-Fo. The Fo indicates the initial minimal fluorescence emitted from a dark-adapted leaf sample and the Fm is the maximal fluorescence measured during the first saturation pulse after dark adaptation. Fv/Fm of CATDLF5- and CATDLF6-inoculated plants and endophyte-free control plants was ~0.73 however it was ~0.83 in natural control plants (Table 1).

### Net CO_2_ assimilation, stomatal conductance and transpiration rate

Natural control plants had 23% higher net CO_2_ assimilation than that of endophyte-free control plants (Table 1). CATDLF5 and CATDLF6 inoculation did not affect the net CO_2_ assimilation in *C. roseus* plants. Stomatal conductance and transpiration rate were 11.3% and 23.7% higher in natural control plants as compared to endophyte-free control plants, respectively (Table 1). CATDLF5- and CATDLF6-inoculated plants had almost similar stomatal conductance and transpiration rate as compared to endophyte-free control plants.

### Starch and biomass production

As photosynthetic efficiency of natural control plants was higher, accumulation of the primary photosynthetic end product starch and biomass of *C. roseus* plant was found to be higher in natural control plants compared to endophyte-free control plants. Natural control plants had 27% and 56% higher starch and biomass production, respectively as compared to that of endophyte-free control plants (Table 1). CATDLF5- and CATDLF6-inoculated plants had an amount of starch and biomass similar to endophyte-free control plants.

### Growth parameters

To evaluate the effect of endophyte inoculation on the growth of *C. roseus* plant, the number of leaves, siliques, branches and plant height were measured. Natural control plants showed better plant growth than that of endophyte-free control plants and CATDLF5- and CATDLF6-inoculated plants. Number of leaves, siliques, branches and plant height was higher in natural control plants (Table 1). CATDLF5 and CATDLF6 inoculation did not affect the growth of the *C. roseus* plants.

### Expression of vindoline biosynthetic pathway genes

**Figure 3:**
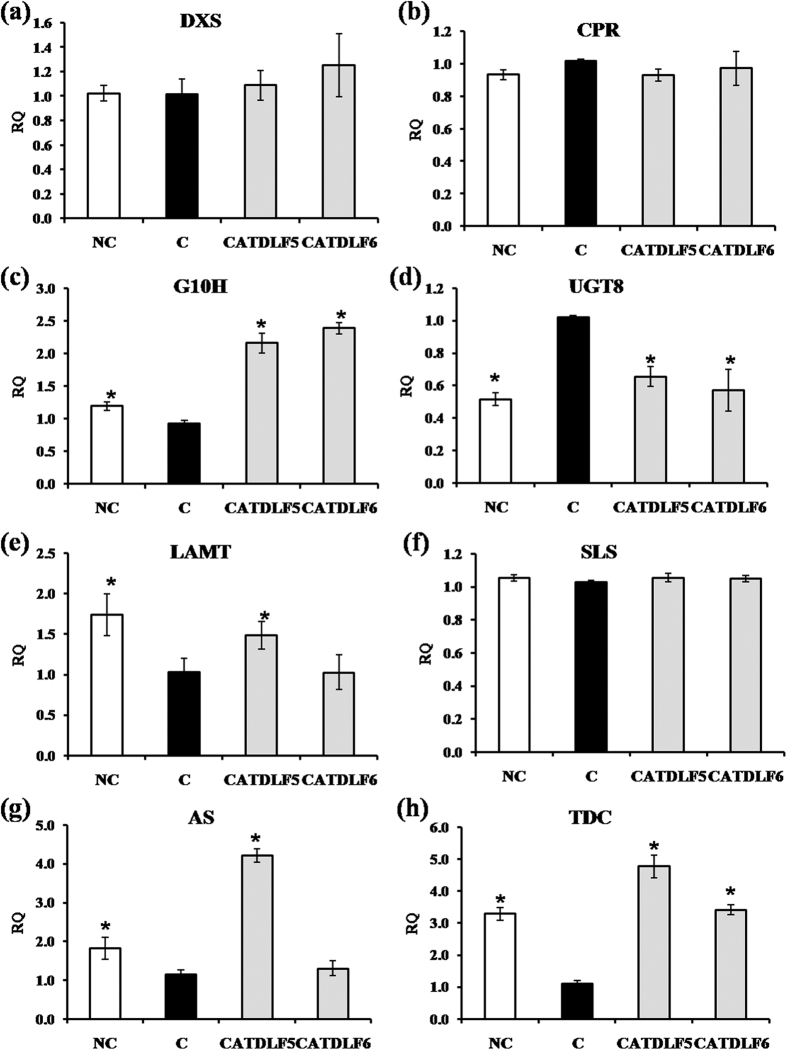
Effect of endophytes (CATDLF5 and CATDLF6) on expression of genes involved in secologanin and tryptamine biosynthesis. Transcript abundance of (**a**) *DXS*, (**b**) *CPR*, (**c**) *G10H*, (**d**) *UGT8*, (**e**) *LAMT*, (**f**) *SLS*, (**g**) *AS*, (**h**) *TDC* was analyzed. NC- the natural control plants that originated from *C. roseus* seeds that were not treated with any bacteriocide and fungicide and contained all the naturally occurring endophytes present in the plants. C- the endophyte-free control plants that originated from *C. roseus* seeds treated with bactericides and fungicides and were thus devoid of any naturally occurring endophyte. The endophyte-free control (C) was used as the calibrator. For normalization, *C. roseus* actin gene was used as the endogenous gene. Data are means ± SD (*n* = 3 biological replicates) and *Y*-axis represents relative quantity (RQ). Asterisks indicate significant differences as compared to the endophyte-free control (C) (Duncan’s multiple range test *P < 0.05).

**Figure 4:**
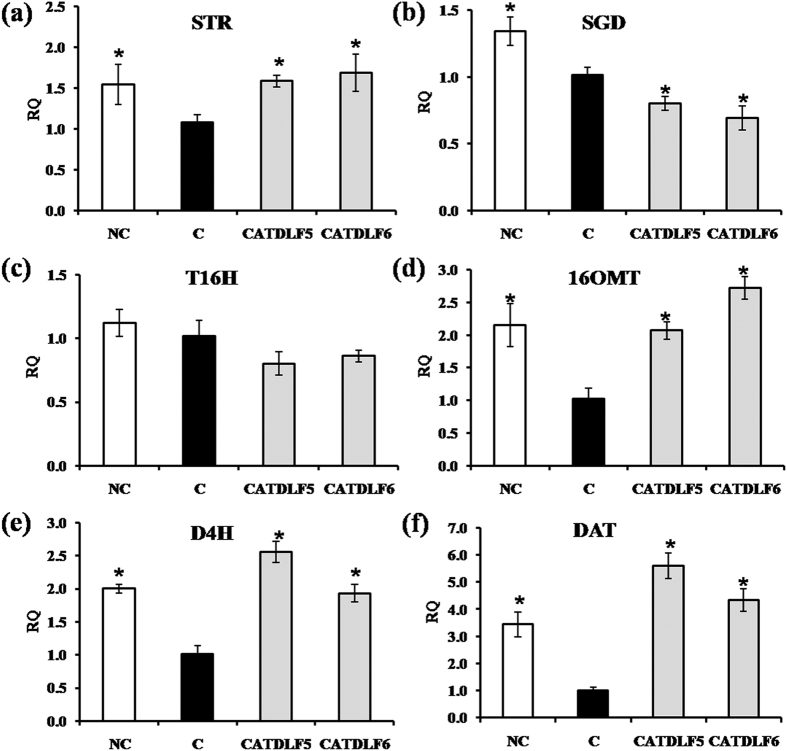
Effect of endophytes (CATDLF5 and CATDLF6) on expression of genes involved in vindoline biosynthesis. Transcript abundance of (**a**) *STR*, (**b**) *SGD*, (**c**) *T16H*, (**d**) *16OMT*, (**e**) *D4H*, (**f**) *DAT* was analyzed. NC- the natural control plants that originated from *C. roseus* seeds that were not treated with any bacteriocide and fungicide and contained all the naturally occurring endophytes present in the plants. C- the endophyte-free control plants that originated from *C. roseus* seeds treated with bactericides and fungicides and were thus devoid of any naturally occurring endophyte. The endophyte-free control (C) was used as the calibrator. For normalization, *C. roseus* actin gene was used as the endogenous gene. Data are means ± SD (*n* = 3 biological replicates) and *Y*-axis represents relative quantity (RQ). Asterisks indicate significant differences as compared to the endophyte-free control (C) (Duncan’s multiple range test *P < 0.05).

To understand the role of CATDLF5 and CATDLF6 in modulation of TIA biosynthesis in the host plant, the expression of different genes involved in TIA biosynthetic pathway was measured by quantitative real time PCR (qRT-PCR) in the third leaves of 90 d-old *C. roseus* plants inoculated individually with CATDLF5 and CATDLF6 endophytes as well as endophyte-free control plants and natural control plants. Endophyte-free control plants were used as the calibrator. Transcript abundance of *CPR*, *DXS*, *G10H*, *UGT8*, *LAMT*, *SLS*, *AS*, *TDC*, *STR*, *SGD*, *T16H*, *16OMT*, *D4H* and *DAT* was quantified (Figs 3 and 4). CATDLF5- and CATDLF6-inoculated and natural control plants had 2–4 fold higher *TDC* expression than that of endophyte-free control plants. Expression of *LAMT* and *AS* was higher in CATDLF5-treated and natural control plants. CATDLF6 did not affect the expression of *LAMT* and *AS*. Expression of *CPR*, *DXS* and *SLS* in the plants were not affected by the presence of endophytes. *UGT8* was downregulated after endophyte treatment. *G10H* expression was enhanced post-endophyte treatment. Expression of *STR*, which catalyzes the condensation of secologanin and tryptamine to produce strictosidine was higher in natural control and CATDLF5- and CATDLF6-inoculated plants. In the case of *SGD*, which codes the protein that catalyzes the first step in the post-strictosidine pathway, the transcript abundance was found to be higher in the natural control plants but lower in the CATDLF5- and CATDLF6-inoculated plants as compared to the endophyte-free control plants. Transcript abundance of other genes for enzymes involved in steps downstream to strictosidine biosynthesis like *16OMT*, *D4H* and *DAT* was found to be higher in CATDLF5- and CATDLF6-inoculated plants as well as the natural control plants as compared to the endophyte-free control plants. Interestingly, there was no significant change in the transcript abundance of *T16H* in the endophyte-inoculated plants and the two types of control (natural and endophyte-free) plants.

### *PRX1* expression and H_2_O_2_ measurement

**Figure 5:**
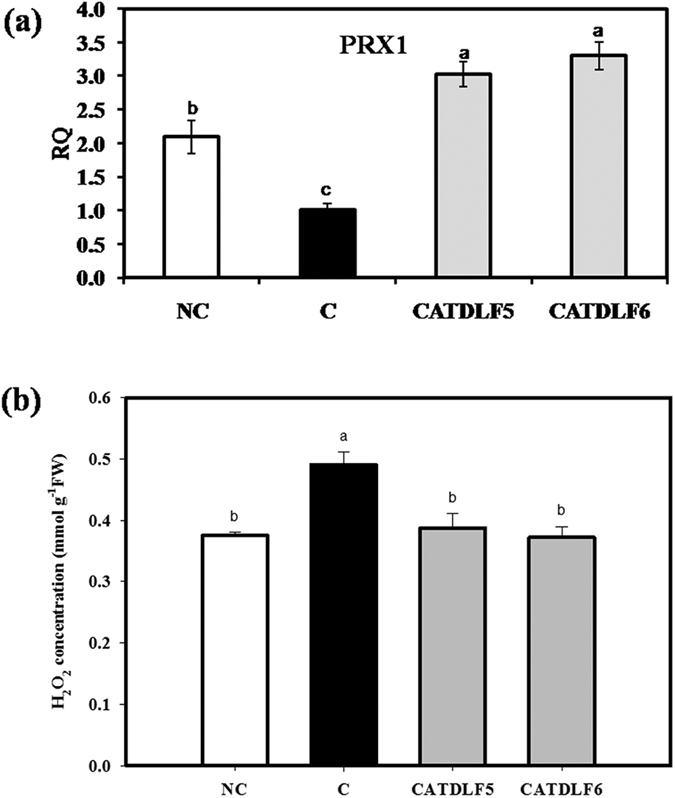
Effect of endophytes (CATDLF5 and CATDLF6) on *PRX1* expression and hydrogen peroxide concentration. (**a**) Transcript abundance of *PRX1*, (**b**) Hydrogen peroxide concentration in the plant leaf. NC- the natural control plants that originated from *C. roseus* seeds that were not treated with any bacteriocide and fungicide and contained all the naturally occurring endophytes present in the plants. C- the endophyte-free control plants that originated from *C. roseus* seeds treated with bactericides and fungicides and were thus devoid of any naturally occurring endophyte. In the qRT-PCR analysis, the endophyte-free control (C) was used as the calibrator and for normalization, *C. roseus* actin gene was used as the endogenous gene. In (**a**) *Y*-axis represents relative quantity (RQ). Data are means ± SD (*n* = 3 biological replicates). Values with different letters are significantly different (Duncan’s multiple range test *P < 0.05).

The expression of class III peroxidase (*PRX1*), which utilizes H_2_O_2_ to oxidize the coupling of vindoline and catharanthine to yield bisindole alkaloids was higher in CATDLF5 and CATDLF6-inoculated and natural control plants as compared to that in endophyte-free control plants (Fig. 5a). H_2_O_2_ production was lower in natural control plants as well as in CATDLF5- and CATDLF6-inoculated plants as compared to endophyte-free control plants (Fig. 5b).

### Regulatory components of TIA biosynthesis

**Figure 6:**
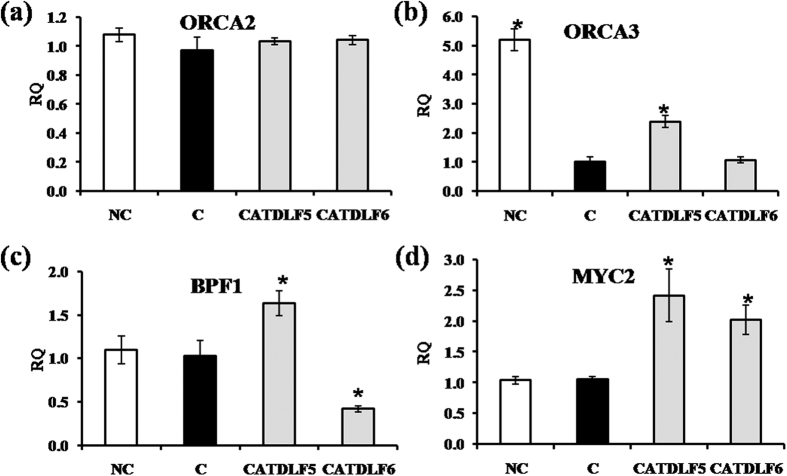
Effect of endophytes (CATDLF5 and CATDLF6) on expression of transcriptional activators. Transcript abundance of (**a**) *ORCA2*, (**b**) *ORCA3*, (**c**) *BPF1*, (**d**) *MYC2* was analyzed. NC- the natural control plants that originated from *C. roseus* seeds that were not treated with any bacteriocide and fungicide and contained all the naturally occurring endophytes present in the plants. C- the endophyte-free control plants that originated from *C. roseus* seeds treated with bactericides and fungicides and were thus devoid of any naturally occurring endophyte. The endophyte-free control (C) was used as the calibrator. For normalization, *C. roseus* actin gene was used as the endogenous gene. Data are means ± SD (*n* = 3 biological replicates) and *Y*-axis represents relative quantity (RQ). Asterisks indicate significant differences as compared to the endophyte-free control (C) (Duncan’s multiple range test *P < 0.05).

**Figure 7:**
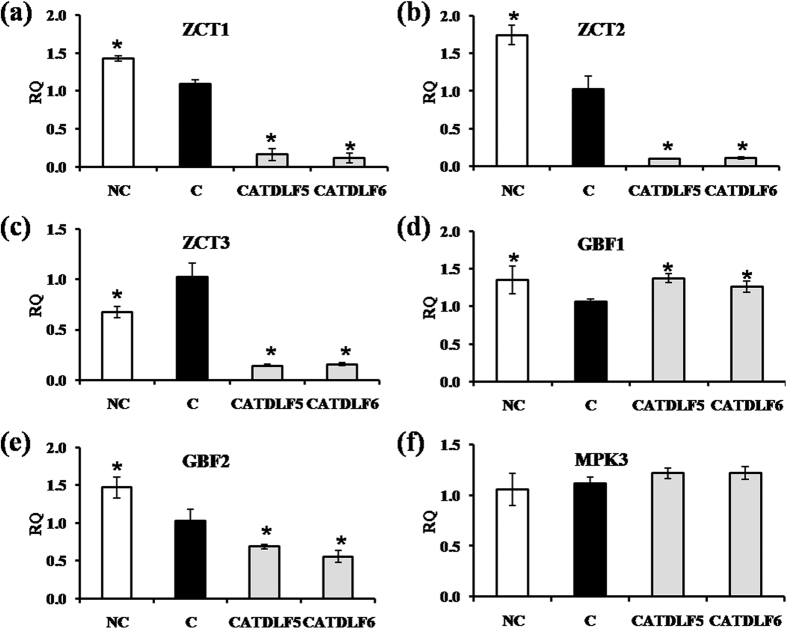
Effect of endophytes (CATDLF5 and CATDLF6) on expression of transcriptional repressors and mitogen activated protein kinase 3. Transcript abundance of (**a**) *ZCT1*, (**b**) *ZCT2*, (**c**) *ZCT3*, (**d**) *GBF1*, (**e**) *GBF2*, (**f**) *MPK3* was analyzed. NC- the natural control plants that originated from *C. roseus* seeds that were not treated with any bacteriocide and fungicide and contained all the naturally occurring endophytes present in the plants. C- the endophyte-free control plants that originated from *C. roseus* seeds treated with bactericides and fungicides and were thus devoid of any naturally occurring endophyte. The endophyte-free control (C) was used as the calibrator. For normalization actin gene was used as the endogenous gene. Data are means ± SD (*n* = 3 biological replicates) and *Y*-axis represents relative quantity (RQ). Asterisks indicate significant differences as compared to the endophyte-free control (C) (Duncan’s multiple range test *P < 0.05).

Since TIA biosynthesis is regulated by transcriptional activators (ORCA2, ORCA3, BPF1 and MYC2) (Fig. 6) and repressors (ZCT1, ZCT2, ZCT3, GBF1 and GBF2) (Fig. 7), their transcript abundance was quantified to understand the mechanism of endophyte-mediated increment in vindoline biosynthesis. *ORCA2* expression was not affected in CATDLF5- and CATDLF6-inoculated plants. When compared to endophyte-free plants, CATDLF5 inoculation enhanced the expression of *ORCA3*, *BPF1*, and *MYC2*, but CATDLF6 inoculation could enhance *MYC2* expression only. Also, CATDLF6 downregulated *BPF1* expression. In natural control plants, *ORCA3* expression was significantly (~5 fold) higher than that in endophyte-free control plants whereas there was no significant difference in expression of *ORCA2*, *BPF1*, and *MYC2* in both types of control (natural and endophyte-free) plants. CATDLF5 and CATDLF6 inoculation downregulated the expression of *ZCT1*, *ZCT2*, *ZCT3* and *GBF2* as compared to the endophyte-free control plants. Unexpectedly, *GBF1* expression was higher in CATDLF5- and CATDLF6-inoculated plants as compared to that in the endophyte-free control plants. Natural control plants showed increased *ZCT1*, *ZCT2*, *GBF1* and *GBF2* expression and decreased *ZCT3* expression as compared to that in the endophyte-free control plants. Transcript abundance of *MPK3*, which has a possible role in the stress-induced biosynthesis of TIAs, was unaffected by CATDLF5 and CATDLF6 inoculation (Fig. 7).

## Discussion

Fungal endophytes have been isolated from *C. roseus* plants and their host and tissue specificity was suggested^34^. A fungal endophyte isolated from *C. roseus* plant was able to produce vinblastine and vincristine in culture medium^10^. Previously, we reported two bacterial endophytes V1 and V3 identified as *Staphylococcus sciuri* and *Micrococcus* sp., respectively isolated from *C. roseus* plants that had increased ~38–68% vindoline content and also enhanced the biomass and plant growth^32^. However, in the present work, we identified two fungal endophytes CATDLF5 (*Curvularia* sp.) and CATDLF6 (*Choanephora infundibulifera*) from alkaloid-rich genotype Dhawal that were able to increase vindoline content by 229–403% in low alkaloid genotype Prabal. We tried to reveal the role of the endophytes in enhancing the biosynthesis of key TIA (vindoline) in the host plant. The endophytes were isolated from field-grown *C. roseus* plants and then their ability to enhance vindoline content in *C. roseus* plants was examined by inoculating them into endophyte-free plants. To have a comprehensive picture, we used two types of control (endophyte-free and natural) plants in the present study. However, the presence of cryptic (non-culturable) endophytes in the endophyte-free plants that were generated for the study could not be completely ruled out. Nevertheless, the effect of any non-culturable microbe present both in the endophyte-free control as well as endophyte (CATDLF5 and CATDLF6)-inoculated plants will not matter as the only variable will be the presence of the additionally inoculated endophyte (CATDLF5 or CATDLF6) when comparing the endophyte-free control with the endophyte-treated plants. Natural control plants, which have all the natural complement of cryptic and non-cryptic endophytes were also used and compared with endophyte-free control plants as well as CATDLF5- and CATDLF6-inoculated plants.

Although the endophytes CATDLF5 and CATDLF6 were able to substantially enhance the vindoline content in the *C. roseus* plant, they did not affect the photosynthetic efficiency of the host plant, which was measured in terms of chlorophyll content and fluorescence, net CO_2_ assimilation, stomatal conductance and transpiration rate. The chlorophyll fluorescence was used as a non-invasive tool to monitor plant performance^35^. Fv/Fm measurement confirmed that natural control plants had more efficient and functional photosynthetic machinery as evident from higher Fv/Fm value in them as compared to that of endophyte-free control plants indicating the possible role of some endophytes in plant physiological processes. Also, higher chlorophyll content in natural control plants may be a reason for higher Fv/Fm value. CATDLF5 and CATDLF6 did not affect the efficiency of photosynthetic apparatus, and the plants inoculated with them had a Fv/Fm value (~0.75) similar to that of endophyte-free control plants. As Fv/Fm value was higher in natural control plants their net CO_2_ assimilation, transpiration rate and stomatal conductance was also higher than that of endophyte-free control plants. The final end product of photosynthetic metabolism is starch, and its accumulation depends on the photosynthetic efficiency of plants^36^. Natural control plants showed higher starch accumulation. Better foliage growth and enhanced biomass of the natural control plants compared to endophyte-free control plants suggests the presence of endophytes that can promote photosynthesis and growth of the host plant. Previously we have reported a bacterial endophyte V3, a *Micrococcus* sp., isolated from *C. roseus* that was able to promote plant growth and biomass^32^. Endophytic fungi that promote photosynthetic efficiency, growth and yield of the plant have also been reported for *Mentha piperita*^37^. A root fungal endophyte *Piriformospora indica* was able to promote plant growth and yield^38^. In future, such endophytes promoting plant growth could be considered for forming a consortium with endophytes enhancing secondary metabolites.

Induction of vindoline biosynthesis by selected endophytes could be the result of upregulation of key structural genes of the TIA biosynthetic pathway such as *G10H*, *LAMT*, *AS*, *TDC*, *STR*, *16OMT*, *D4H* and *DAT*. However, it seems that each endophyte differentially regulates the expression of a different set of genes involved in TIA biosynthesis. Both the endophytes also upregulated the expression of vacuolar localized *C. roseus* class III peroxidase (*PRX1*), which dimerizes the monomeric TIA precursors (vindoline and catharanthine) to form bisindole alkaloids^39^. PRX1 is also known to be involved in scavenging of H_2_O_2_ and its homeostasis in plant cell^40^. As suggested previously, PRX1-secondary metabolites together serve as a central sink and buffer of H_2_O_2_ levels in plant cells and fine-regulation of this system is needed for efficient TIA production and plant homeostasis^40^. However, there may be further complexities involved as well. Higher *PRX1* expression in CATDLF5- and CATDLF6-inoculated plants as compared to natural control plants could not result into lower H_2_O_2_ accumulation, whereas higher *PRX1* expression in natural control plants compared to endophyte-free control plants could reduce the accumulation of H_2_O_2_ but could not increase the vindoline content as in the case of CATDLF5- and CATDLF6-inoculated plants. Although vindoline is a substrate for PRX1, there seems to be no direct relationship between *PRX1* expression and vindoline content in the present study. In natural control plants, lower H_2_O_2_ production indicates the presence of some endophytes in host plant that can scavenge H_2_O_2_ or having antioxidative properties. Endophytes promoting the antioxidative potential of host plants have been isolated from *Datura stramonium*^41^ and *Solanum nigrum*^42^.

Biosynthesis of TIA is regulated by transcriptional regulators. Three Cys2/His2-type zinc finger protein family transcriptional factors ZCT1, ZCT2 and ZCT3, have been identified to repress the activity of *TDC* and *STR* promoters^43^. ZCTs also repress the activating activity of ORCAs, APETALA2/ethylene response factor domain transcription factors^43^. Transcriptional activities of *TDC*, *STR* and *D4H* promoters are also regulated by ORCA2 and ORCA3^44^,^45^. Downregulation of *ZCT* expression (in CATDLF5- and CATDLF6-inoculated plants) and upregulation of *ORCA3* expression (in CATDLF5-inoculated plants only) may be the reason for higher *TDC* and *STR* transcript abundance. Previously it was found that transgenic plants overexpressing *ORCA3* had induced expression of *TDC*, *STR*, *AS*, and *D4H*^46^. An earlier study^28^ revealed that *ORCA* expression was activated by the basic helix-loop-helix transcription factor CrMYC2. The selected endophytes regulated the expression of *STR* by differentially modulating the expression of two G-box-binding factors *GBF1* and *GBF2* that act as transcriptional repressors of *STR* promoters^47^. Expression of mitogen-activated protein kinase *MPK3* was not affected by selected endophytes, although stresses such as wounding, UV-treatment, and methyl jasmonate application induce *MPK3* expression^48^. This confirmed that the selected endophytes induced *in planta* vindoline biosynthesis without creating any major type of stress response in the host plant.

Results of the present study suggest that the presence of natural endophytes in the plant probably plays an important role in promoting the growth of the host plant by increasing photosynthetic pigment synthesis, photosynthetic rate, stomatal conductance, transpiration rate, which results in increased starch accumulation and biomass. The absence of these natural endophytes (in endophyte-free plants) resulted in reduced growth parameters. Our study strongly suggests that the presence of some endophytes is able to promote the biosynthetic potential of the plant for some key secondary metabolites. Two such endophytes (CATDLF5 and CATDLF6) could enhance vindoline content by modulating the expression of genes involved in TIA biosynthesis and its regulatory components. For future applications, we suggest formulation and testing of microbial consortia comprised of endophytes like bacterial V3 (that enhance plant growth rate, biomass and vindoline content) and fungal CATDLF5/CATDLF6 (that enhance vindoline content appreciably but have no effect on plant growth/biomass). Such consortia may provide a win-win situation by providing high biomass coupled with high vindoline content, which in turn might play a key role in reducing the price of the costly bisindole alkaloids.

## Methods

### Plant material and growth conditions

Seeds of two *C. roseus* genotypes (cv. Prabal and cv. Dhawal) were obtained from the National Gene Bank for Medicinal and Aromatic Plants at CSIR-CIMAP, Lucknow and grown in pots (17 cm height ×22 cm top diameter ×12 cm bottom diameter and 3.7 L volume) filled with 3.5 kg of autoclaved soil and vermicompost mixture (2:1 v/v). The plants were watered with sterile water. Plants were grown in a greenhouse in natural photoperiod and light intensity at 25 °C ± 2 °C.

### Isolation of endophytes

Endophytes were isolated from healthy green leaves of field grown *C. roseus* (cv. Dhawal) plants. For surface sterilization leaves were washed in running tap water and then dipped in 1% sodium hypochlorite for 10 min and rinsed 4 times in 0.02 M sterile potassium phosphate buffer pH 7.0. Sterility check was performed by taking 100 μL of an aliquot from the final wash and transferring to 5 mL nutrient broth and keeping in an incubator shaker (200 rpm at 28 °C) for 7 days. Samples were discarded if the growth was observed in the sterility check samples. Surface sterilized leaves were cut into small pieces by using sterilized surgical blade and kept on potato dextrose agar (PDA) (200 g L^−1^ potato infusion, 20 g L^−1^ dextrose, 15 g L^−1^ agar; pH 5.6) plate. Sterilized leaves were also macerated in a sterile pestle and mortar with sterile distilled water. The extract (100 μL) was taken and serial dilutions were made. Each dilution of the sample was plated on PDA plate. The plates were incubated at 28 °C for 7 days. Emerged fungal colonies were separated under sterilized conditions and grown on PDA plates for further use. Isolated fungal endophytes were identified by sequencing the internal transcribed spacer (ITS; 18S rDNA) region.

### Fungal ITS fragment amplification and sequencing

Genomic DNA from endophytes was isolated by using CTAB method^49^. Primers ITS1 (5′-TCCGTAGGTGAACCTGCGG-3′) and ITS4 (5′-TCCTCCGCTTATTGATATGC-3′) used for PCR amplification of the ITS were taken from a previous study^50^. Each PCR reaction mixture (25 μL) contained 1 μL genomic DNA (100 ng), 1 μL each of 10 μM forward and reverse primers, 0.5 μL 10 mM deoxyribonucleotide triphosphate mixture, 2.5 μL 10X PCR buffer (100 mM Tris-HCl, pH 8.3; 500 mM KCl; 15 mM MgCl_2_; 0.01% gelatin), 0.2 μL Taq DNA polymerase (5 U μL^−1^) (Sigma-Aldrich Inc. MO, USA), and 18.8 μL autoclaved MilliQ water. The thermocycling conditions consisted of an initial denaturation step of 94 °C for 5 min, followed by 30 cycles of 94 °C for 45 s, 57.4 °C for 45 s, and 72 °C for 2 min, and a final extension at 72 °C for 5 min in an Eppendorf Master cycler gradient PCR system. PCR products were visualized on 0.8% agarose gels, and the products were excised and purified with Nucleo-pore PCR Clean-up Gel Extraction kit (Genetix Biotech Asia Pvt. Ltd., India) following the manufacturer’s instructions. DNA sequencing was performed with a 3130XL genetic analyzer (Applied Biosystems, USA). Nucleotide sequence similarities were determined by using NCBI BLAST search (http://blast.ncbi.nlm.nih.gov/blast/Blast.cgi). Partial sequence data for the ITS fragment have been deposited in the GenBank (NCBI) under accession numbers KT001517 (CATDLF5) and KT001518 (CATDLF6).

### Treatment of *C. roseus* plants with endophytes

Endophyte-free *C. roseus* (cv. Prabal) plants were used to study the effects of treatment with endophytes isolated from cv. Dhawal. Endophyte-free plants were generated according to an earlier report^51^. Seeds were rinsed thoroughly with water and incubated in fungicide Bavistin (containing carbendazim 50% W.P., BASF India Limited) and bactericide K-Cycline (containing tetracycline hydrochloride 10% w/w and streptomycin sulphate 90% w/w, Karnataka Antibiotics & Pharmaceuticals Ltd. Bangalore, India) solution at room temperature with shaking at 100 rpm. Carbendazim, tetracycline hydrochloride and streptomycin sulphate were chosen because no phytotoxic effects were observed on the treated seedlings. After 24 h of treatment, seeds were washed five times with autoclaved water and an aliquot of seeds was crushed using a sterile pestle and mortar with sterile distilled water. Triturate was plated on nutrient agar and potato dextrose agar and plates were incubated at 28 °C for 10 days. When no microbial growth was found in the incubated plates, the remaining seeds were treated as endophyte-free and were used to grow endophyte-free nursery stock of *C. roseus* in pots (7 cm height ×30 cm top diameter ×20 cm bottom diameter and 3.0 L volume) filled with 3.0 kg of autoclaved soil and vermicompost mixture (2:1 v/v) and watered with sterile water. Pots were maintained in a greenhouse (natural photoperiod and light intensity at 25 °C ± 2 °C) for one month and sample seedlings were re-checked for their endophyte-free status. Thirty days-old endophyte-free seedlings were uprooted carefully to minimize damage to their roots and used for inoculation with the selected/target endophytes. For inoculation, the roots of the seedlings were dipped in individual endophyte spore suspension (1 × 10^8^ spore/conidia mL^−1^) prepared in phosphate buffered saline (PBS) (8 g L^−1^ sodium chloride, 0.2 g L^−1^ potassium chloride, 1.44 g L^−1^ disodium hydrogen phosphate, 0.24 g L^−1^ potassium dihydrogen phosphate; pH 7.4) for 3 h. The plants were re-planted in autoclaved soil and vermicompost mixture (2:1 v/v) filled pots (17 cm height ×22 cm top diameter ×12 cm bottom diameter and 3.7 L volume) and grown under greenhouse conditions. Plants were watered with sterile water as and when required.

For all experimental analyses, endophyte-treated plants were compared with two types of controls-(i) the endophyte-free control [C] plants that originated from *C. roseus* seeds treated with bactericides and fungicides and were thus devoid of any naturally occurring endophyte and (ii) the natural control [NC] plants that originated from *C. roseus* seeds that were not treated with any bacteriocide and fungicide and contained all the naturally occurring endophytes present in the plants. The purpose to include the natural control was to get an idea of the effect of the natural endophytic populations occurring in the plants *vis-à-vis* the endophyte-free control plants. The roots of both the controls–endophyte-free (C) and natural (NC) plants were dipped in PBS for 3 h before re-planting in autoclaved soil and vermicompost mixture (2:1 v/v) filled pots. To ensure the presence of a sufficient number of inoculated endophytes in the soil, the inoculation (10 mL pot^−1^ of the individual endophyte suspension containing 1 × 10^8^ spore/conidia mL^−1^) was repeated at 15-days after the first inoculation. Before carrying out further experimental analyses, the presence of endophytes in the inoculated *C. roseus* plants was confirmed by incubation of small pieces of surface- sterilized leaves on PDA plate. Inoculated endophytes were found to emerge from the margin of pieces cut from the leaves of endophyte-inoculated plants.

To minimize variations related to plant developmental stage and other variables, sampling for all analyses were carried out at the same (intermediate-neither too young nor too old/senescent) stage (90 d) and position of leaves (third leaf from top).

### Analysis of vindoline content

Extraction of *C. roseus* leaves for alkaloid analysis was done according to an earlier report^52^. It is known that vindoline content depends upon plant developmental stage^20^. Hence, sampling was carried out in plants of the same stage (90-d old) and from the same position of leaves (third leaf) in each case to minimize unintended variations. Leaf powder (1 g) was extracted thrice with 30 mL of 90% ethanol for 12 h at room temperature. The alcohol extract was filtered through Whatman No. 1 filter paper. The filtrates were concentrated in vacuo (Rotavapor R-210, Büchi, Switzerland) to 10 mL, diluted with water (10 mL), acidified with 3% HCl (10 mL) and washed with hexane three times (3 × 30 mL). The aqueous portion was separated and basified with liquid ammonia to pH 8.5 and extracted three times with chloroform (3 × 30 mL). The chloroform extract was washed with water, dried over anhydrous sodium sulphate, concentrated under vacuum and redissolved in 1 mL methanol. Vindoline content in the methanol extract of leaf tissue was measured by using HPLC as described previously^53^. In brief, separation was achieved on C_18_ Symmetry® Waters (250 × 4.6 mm, 5μm) using the mobile phase consisting 70:30 (v/v) mixture of 100 mM ammonium acetate (pH 7.3) (A): acetonitrile (B), at a flow rate of 1 mL min^−1^ for 5 min. The mobile phase was changed in a linear gradient to 36A:64B over 10 min and maintained for 15 min. The flow rate was increased to 1.4 mL min^−1^ over 5 min. The A:B ratio was then increased to 20:80 in 5 min and maintained for 15 min. The flow rate and mobile phase ratio were then returned to 1 mL min^−1^ and 70:30 and the column was allowed to re-equilibriate. The analysis was performed with Empower Pro software (Waters, Milford, MA, USA). Pure vindoline (Sigma-Aldrich, St. Louis, MO, USA) was used as a standard. The selectivity and specificity of the vindoline determination was established through-(i) studying peak purity plots using a PDA detector, (ii) UV-Vis spectra matching, (iii) spiking with reference compound of known concentration, and (iv) LC-MS specificity (Supplementary Figs S3 and S4).

### Photosynthetic pigments, chlorophyll fluorescence and photosynthesis measurement

Total chlorophyll, chlorophyll a, chlorophyll b and carotenoid content was measured in the plants. Leaf tissue was kept overnight in 100% methanol at 4 °C and quantified as described previously^54^.

Chlorophyll fluorescence and photosynthesis in terms of net CO_2_ assimilation, stomatal conductance, and transpiration rate were measured in the attached plant leaf using portable photosynthesis system (CIRAS-3 PP Systems, USA) attached with chlorophyll fluorescence module (CFM-3). CO_2_ concentration in sample chamber was maintained at 400 ppm (ambient CO_2_), and chamber air temperature was maintained at 25 °C (ambient temperature). Leaf was incubated in dark for 15 min before chlorophyll fluorescence measurement. For CO_2_ assimilation, stomatal conductance and transpiration rate measurement, the leaf was pre-exposed for 15 min at 400 μmol photons m^−2^ s^−1^ light, 400 ppm CO_2_, and 25 °C temperature.

### Starch estimation

Starch content was estimated in the leaves by the acid digestion method as described previously^55^. Leaf discs of equal diameter were homogenized in liquid nitrogen. These homogenized samples were washed with acetone and hot 80% ethanol to remove interfering substances until the extract became colorless. The starch was extracted and solubilized by 35% perchloric acid. This starch solution was used for the colorimetric assay by using anthrone reagent (1.146 g of anthrone powder in 500 mL of concentrated sulfuric acid and 200 mL of water). Samples were placed in a boiling-water bath for 12 min and immediately placed in ice. Absorbance was recorded at 625 nm on a spectrophotometer.

### Hydrogen peroxide estimation

Hydrogen peroxide was determined in the plant leaves as described previously^56^ with slight modifications. Leaf tissues (200 mg) were homogenized in 1 mL of 0.1% (w/v) trichloroacetic acid solution on ice. The homogenate was centrifuged at 12,000 g for 15 min at 4 °C, and 0.4 mL of the supernatant was then added to 0.4 mL of 10 mM potassium phosphate buffer, pH 7.0, and 0.8 mL of 1 M potassium iodide and mixed. The absorbance was measured at 390 nm by using a spectrophotometer. The content of H_2_O_2_ was calculated by comparison with a H_2_O_2_ calibration curve.

### Measurement of plant biomass, plant height, number of leaves, branches and siliques

For biomass measurement, aerial parts of the plants were harvested and dried at 70 °C for 5 d and their dry weights were measured. Plant height, number of leaves, siliques and branches were also measured.

### qRT-PCR analysis

Total RNA was extracted from the leaves using TRI-reagent (Sigma-Aldrich, St. Louis, USA) according to the manufacturer’s instructions. RNA quantification was performed by using NanoDrop 1000 spectrophotometer (Thermo Fisher Scientific). To remove genomic DNA contamination, DNase I, RNase-free enzyme (Thermo Scientific) was used. First-strand cDNA synthesis was done from 5 μg of total RNA using RevertAid First Strand cDNA Synthesis Kit (Thermo Scientific) with oligo(dT)_18_ primer according to the manufacturer’s protocol. Transcript abundance of structural and regulatory genes involved in vindoline biosynthesis was analyzed. Primers used for the qRT-PCR were designed using Primer Express Software v3.0.1 (Applied Biosystems) (Supplementary Table S1)^48^,^57^,^58^. PCR mixtures included 5 μL Power SYBR Green PCR Master Mix (Applied Biosystems, Warrington, UK), 300 nM each of the forward and reverse primers and 1 μL of 10 times diluted cDNA as a template in a reaction volume of 10 μL. PCR conditions were an initial denaturation of 10 min at 95 °C, followed by 40 cycles of denaturation at 95 °C for 15 sec and annealing/extension at 60 °C for 1 min each. Fluorescent signal intensities were recorded on an Applied Biosystems StepOnePlus^TM^ Real-Time PCR System and analyzed using StepOne software (Applied Biosystems). Specificity of qRT-PCR was examined by subjecting all amplicons to a melting curve analysis using the dissociation method (Applied Biosystems). The threshold cycle (Ct) for each gene was normalized against the Ct for actin of *C. roseus*, which was used as the endogenous gene. The endophyte-free control (C) was used as the calibrator and the relative quantification 2^−∆∆Ct^ method was used^59^,^60^.

### Statistical analysis

For each treatment (endophytes and controls) three individual plants were analyzed for qRT-PCR, vindoline content, and hydrogen peroxide measurement whereas for analysis of physiological and growth parameters six individual plants were analyzed as biological replicates. For each biological replicate, three technical replicates were run to keep a check on analytical errors and the mean of the three technical replicates represented the particular biological replicate. Technical replicates were carried out for qRT-PCR, vindoline content, and hydrogen peroxide measurement but not for physiological and growth parameters analyses. Statistical analyses were carried out for the data obtained for the three biological replicates by applying ANOVA, suitable for a completely randomized design (CRD), using the ASSISTAT Version 7.6 beta (2012) software. Comparisons among means were carried out using Duncan’s multiple range tests at a significance level of P ≤ 0.05.

## Additional Information

**How to cite this article**: Pandey, S. S. *et al.* Fungal endophytes of *Catharanthus roseus* enhance vindoline content by modulating structural and regulatory genes related to terpenoid indole alkaloid biosynthesis. *Sci. Rep.* **6**, 26583; doi: 10.1038/srep26583 (2016).

## Supplementary Material

Supplementary Information

## Figures and Tables

**Figure 1:**
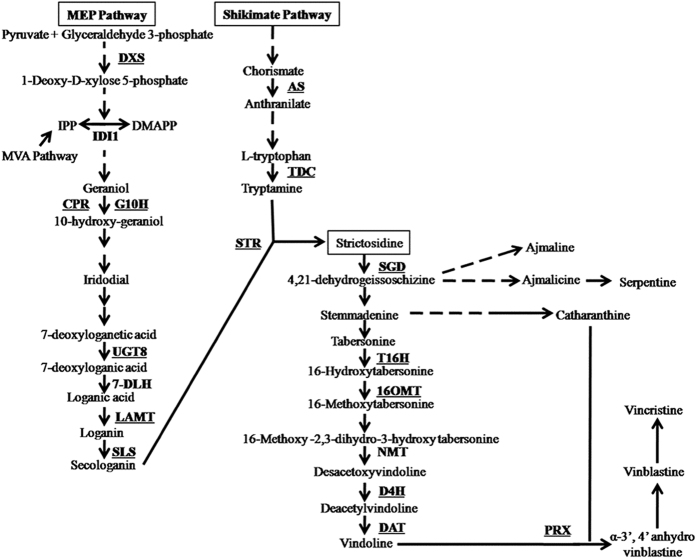
Schematic representation of the terpenoid indole alkaloid (TIA) biosynthetic pathway. *Enzyme abbreviations:* DXS, 1-deoxy-D-xylulose-5-phosphate synthase; IPP, isopentenyl pyrophosphate; DMAPP, dimethylallyl pyrophosphate; IDI1, isopentenyl diphosphate isomerase 1; CPR, cytochrome P450 reductase; G10H, geraniol 10-hydroxylase; UGT8, UDP-sugar glucosyltransferase 8, 7-DLH, 7-deoxyloganic acid hydroxylase; LAMT, loganic acid *O*-methyltransferase; SLS, secologanin synthase; AS, anthranilate synthase; TDC, tryptophan decarboxylase; STR, strictosidine synthase; SGD, strictosidine glucosidase; T16H, tabersonine 16-hydroxylase; 16OMT, 16-hydoxytabersonine-*O*-methyltransferase; NMT, *N*-methyltransferase; D4H, desacetoxyvindoline-4-hydroxylase; DAT, deacetylvindoline-4-*O*-acetyltransferase; and PRX, vacuolar class III peroxidase.

**Table 1:** Effect of inoculation with endophytes (CATDLF5 and CATDLF6) on physiological and growth parameters of *Catharanthus roseus* plants.

Treatment	NC	C	CATDLF5	CATDLF6
Chlorophyll (mg gFW^−1^)	1.74 ± 0.03^a^	1.55 ± 0.03^b^	1.61 ± 0.04^b^	1.55 ± 0.03^b^
Carotenoids (mg gFW^−1^)	0.07 ± 0.010^a^	0.06 ± 0.005^a^	0.06 ± 0.003^a^	0.07 ± 0.004^a^
Fv/Fm	0.83 ± 0.01^a^	0.73 ± 0.01^b^	0.73 ± 0.02^b^	0.75 ± 0.02^b^
A (μmol m^−2^ s^−1^)	14.77 ± 0.43^a^	11.97 ± 0.20^b^	12.47 ± 0.60^b^	11.70 ± 0.71^b^
E (mmol m^−2^ s^−1^)	10.63 ± 0.46^a^	8.59 ± 0.41^b^	8.91 ± 0.17^b^	8.81 ± 0.44^b^
gS (mmol m^−2^ s^−1^)	481.00 ± 5.13^a^	432.00 ± 8.02^b^	432.33 ± 12.12^b^	410.70 ± 10.68^b^
Starch (mmol m^−2^)	41.35 ± 0.56^a^	32.45 ± 1.43^b^	32.19 ± 3.42^b^	30.54 ± 2.04^b^
Biomass (g Plant^−1^)	5.03 ± 0.40^a^	3.23 ± 0.22^b^	3.14 ± 0.10^b^	3.27 ± 0.23^b^
Number of leaves (Plant^−1^)	75.33 ± 2.90^a^	58.67 ± 3.52^b^	59.33 ± 4.37^b^	55.00 ± 2.60^b^
Number of siliques (Plant^−1^)	23.00 ± 1.15^a^	16.00 ± 1.15^b^	17.33 ± 1.20^b^	15.67 ± 1.45^b^
Number of branches (Plant^−1^)	5.67 ± 0.27^a^	4.33 ± 0.27^b^	4.33 ± 0.13^b^	4.67 ± 0.13^b^
Plant height (cm)	39.67 ± 1.20^a^	35.67 ± 0.33^b^	36.00 ± 1.31^b^	33.33 ± 2.40^b^

NC- the natural control plants that originated from *C. roseus* seeds that were not treated with any bacteriocide and fungicide and contained all the naturally occurring endophytes present in the plants.

C- the endophyte-free control plants that originated from *C. roseus* seeds treated with bactericides and fungicides and were thus devoid of any naturally occurring endophyte. Fv/Fm indicates the quantum efficiency of photosynthesis, A-Net CO_2_ assimilation, E-Transpiration rate, gS-Stomatal conductance.

Values are the means of six biological replicates ± S.E. Values with different letters (a, b) are significantly different at *P* ≤ 0.05 (Duncan’s multiple range test).
