# Supplementary material for: Fungal endophytes of Catharanthus roseus enhance vindoline content by modulating structural and regulatory genes related to terpenoid indole alkaloid biosynthesis
Source: Sci Rep. 2016 May 25;6:26583. doi: 10.1038/srep26583 (PMC4879578; doi:10.1038/srep26583)
Supplement: Supplementary Information [file srep26583-s1.pdf]

Supplementary Material

Fungal endophytes of *Catharanthus roseus* enhance vindoline content by modulating structural and regulatory genes related to terpenoid indole alkaloid biosynthesis

Shiv S Pandey, Sucheta Singh, CS Vivek Babu, Karuna Shanker, N K Srivastava, Ashutosh K Shukla, Alok Kalra

Supplementary Table S1 List of primers used for quantitative real time PCR analysis

Genes	Primers	5'-3'
ORCA3	Forward	CCGGACCCGTTAGAGTAAACC
	Reverse	CGTCTCTTCTTCCTTCCTCCAC
G10H	Forward	GGTAGCCTCACGATGGAGAA
	Reverse	CCTTGGCAGAATCCGAATAA
DXS	Forward	TCGCTGCAGAACTTAGAGCA
	Reverse	GCCAACATCCCAAATGATTC
TDC	Forward	CGCCTGTATATGTCCCGAGT
	Reverse	GTTGCGATTTGCCAATTTTT
STR	Forward	TGCCACACAACTAGCCACAA
	Reverse	TCATGATTTCTTCCACACCTTCG
D4H	Forward	TACCCTGCATGCCCTCAACCA
	Reverse	AGAGCTCCAGGAATGAAGGGG
MYC2	Forward	TTTGGCAGTCGTCTGTTGTC
	Reverse	TCGGTATCGGTCACCTCTTC
AS	Forward	CTACTGTCGCACAACCTAC
	Reverse	CGTCTCATAACTGGCTCA
MPK3	Forward	ACGAAATGAGGATGCAAAAAGATAC
	Reverse	TGCTAACTGCTGACGAGGGAAT
DAT	Forward	AATCCCTCAGCCGCTATAACC
	Reverse	ACGGATACGCACGTTTGGTAT
ZCT1	Forward	CATGGGCGTGAAGAGATTCA
	Reverse	CCGACTTTAGAAAGAAGCATCAAAC
ZCT2	Forward	CCGATGAAGCGTACGAGAGAA
	Reverse	TCAAGCAATTCGCCATAGTTGT
ZCT3	Forward	CACCTACACCTGTTTTTCAATACGA
	Reverse	GCCCATGGCTGATCTAGATAGC
SGD	Forward	TCAGTCCCTTGTTGTTGCCA
	Reverse	ACGATGAACGATGGGCTTGT
T16H	Forward	ATGCCCATCAACTTAGCGGT
	Reverse	AAGTGCATCAACGGCCCATA
16OMT	Forward	TGGATTCTCCATGACTGGAACG
	Reverse	ACCCTTCGCTGGAATTGCTT
PRX1	Forward	GCCCTTGAAAGGGAGTGTCC
	Reverse	TGGCCCTCCTAACGAAACAA
SLS	Forward	GAAAGGCAATTGCTGCCACT
	Reverse	ACCATGCCCAATCCAACACT
Actin	Forward	CTATGTTCCCAGGTATTGCAGATAGA
	Reverse	GCTGCTTGGAGCCAAAGC
UGT8	Forward	CCATGCTCAGACTAGCAGAAC
	Reverse	AAGCGACTGTGGCTGAACTCTG
LAMT	Forward	GAAATGCCTGCTCTTCCAAC
	Reverse	GTGGGAGTCATCACCACCTT

ORCA2	Forward	TCAACAACGATTTTGATTTTTCA
	Reverse	TCCGAAGCATAATTTGGTGA
BPF1	Forward	TGGACCGAGTTTTATCTGCTC
	Reverse	TTCCCGGTTTGCTTAGACTG
GBF1	Forward	CAGAGAAAGCTATGAGGGCAAG
	Reverse	CACCCATCACCTTTTCAGTTG
GBF2	Forward	AGAATCTGCTCGGCGATCTA
	Reverse	CGCTGAGCCAATTCATCA
CPR	Forward	TTGCAGTGAGGAAGGAGCTT
	Reverse	AATCCAAATGGGTGCAAGAA

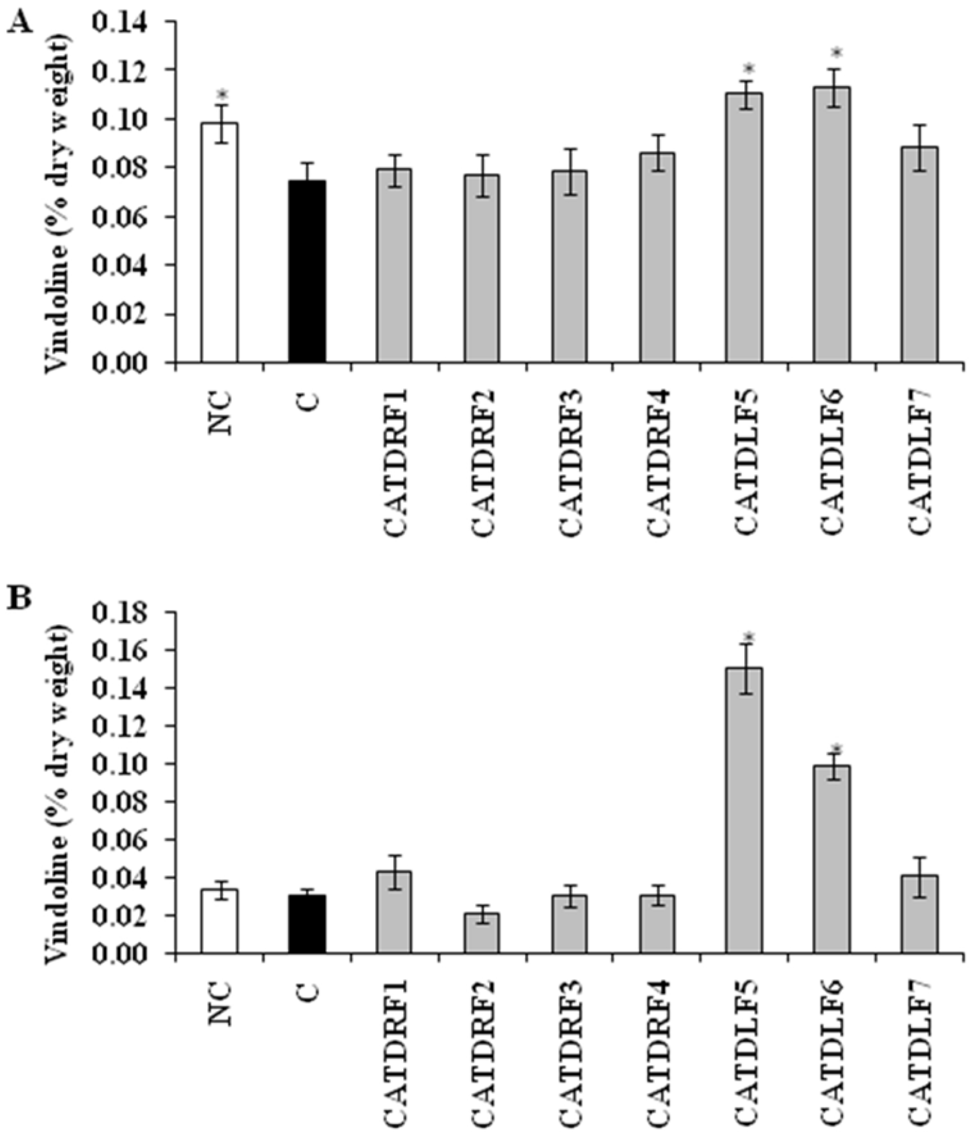

Supplementary Fig. S1: Effect of endophytes on vindoline content of *Catharanthus roseus* leaves. (**A**) Vindoline content in leaves of endophyte inoculated *C. roseus* cv. Dhawal

(**B**) Vindoline content in leaves of endophyte inoculated *C. roseus* cv. Prabal. Endophyte-free *C. roseus* (cv. Dhawal and cv. Prabal) plants (generated from seeds treated with bactericide and fungicide) were used to study the effect of treatment with endophytes (CATDRF1, CATDRF2, CATDRF3, CATDRF4, CATDLF5, CATDLF6 and CATDLF6 isolated from cv. Dhawal) on

leaf vindoline content. Two types of controls were included in the study - (i) the endophyte-free control [C] plants that originated from *C. roseus* seeds treated with bactericides and fungicides and were thus devoid of any naturally occurring endophyte and (ii) the natural control [NC] plants that originated from *C. roseus* seeds that were not treated with any bacteriocide and fungicide and contained all the naturally occurring endophytes present in the plants. Fungal endophyte inoculums (1 x 10^8 spore/conidia mL^{-1}) prepared in phosphate buffer saline (PBS) were used to treat roots of experimental plants. The roots of both the controls – endophyte-free (C) and natural (NC) plants were treated with PBS. Third leaves of 90 d-old plants were sampled for vindoline content (% dry weight basis). As biological replicates, three plants per treatment were analyzed. For each biological replicate, three technical replicates were run on the HPLC and the mean of the three technical replicates represented the particular biological replicate. Statistical analysis was carried out for the data obtained for the three biological replicates (n=3). Asterisks indicate significant differences as compared to the endophyte-free control (C) (Duncan's multiple range test *$P<0.05$)

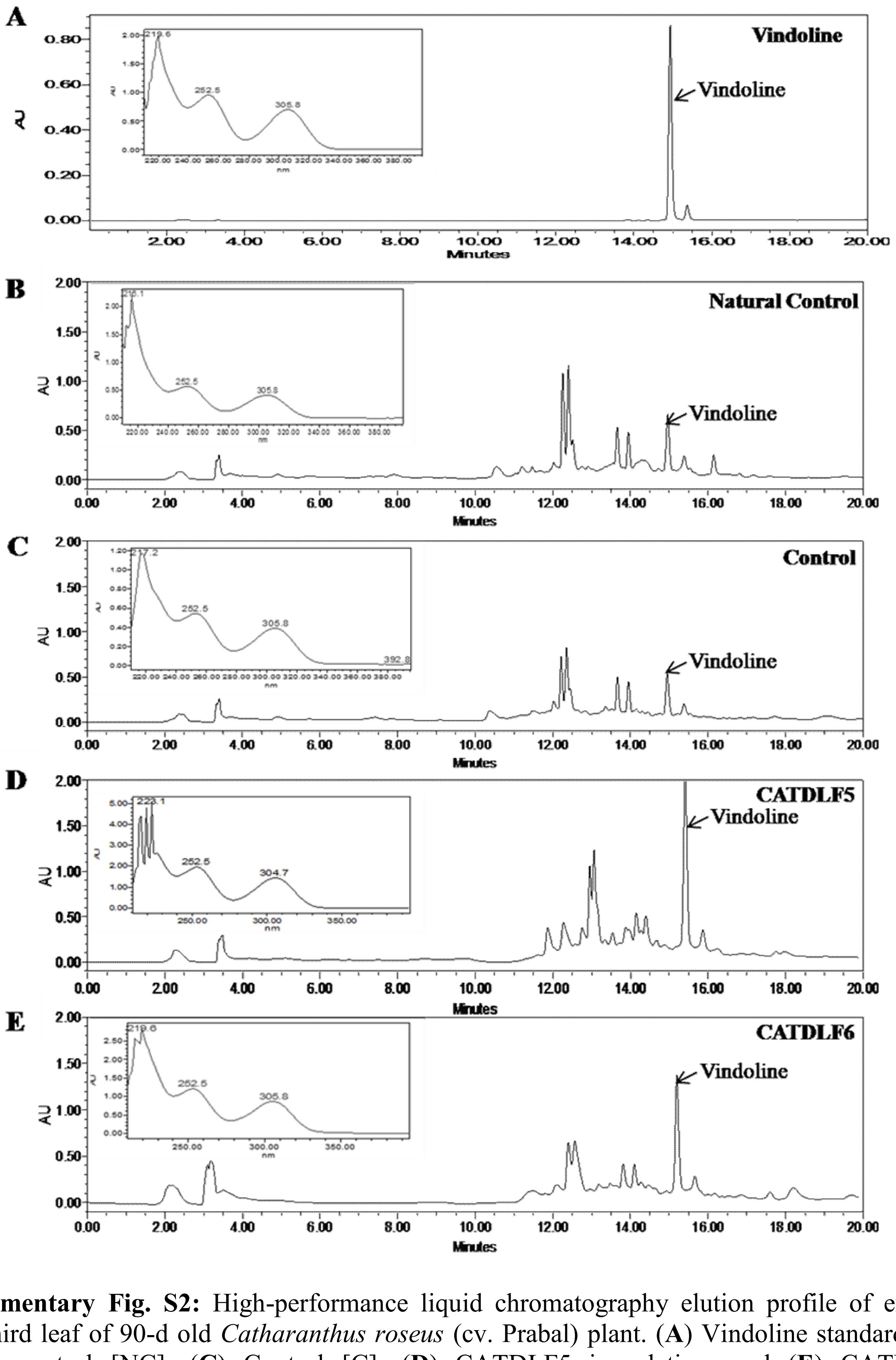

Supplementary Fig. S2: High-performance liquid chromatography elution profile of extract from third leaf of 90-d old *Catharanthus roseus* (cv. Prabal) plant. (**A**) Vindoline standard, (**B**) Natural control [NC], (**C**) Control [C], (**D**) CATDLF5 inoculation, and (**E**) CATDLF6 inoculation. Picture in inset represents UV-profile of vindoline.

Specificity of vindoline determination using LC-MS spectral characteristics and matching

The specificity of vindoline analysis was further ensured by using LC-MS. For this, representative samples were reanalysed using same analytical conditions as used for HPLC (mentioned in the methods section) on Shimadzu LC-MS 2010 EV system (Shimadzu, Japan) comprising of binary pump (LC-20AD), degasser, column oven (CTO-20), rheodyne injector, analytical column (Waters X-select®, 250 × 4.6 mm, 5µm), and controlled by LCMS solution (Version 3). The part of the column out flow (1/4th part) was allowed to the mass detector. The retention time of vindoline was 12.13 min. The molecular mass of the product was determined with an ESI probe in both positive and negative ionization mode. The characteristic ion adduct of vindoline i.e. 457 $[M+H]^+$, 458 $(M+2H]^+$ and 479 $(M+Na]^+$ were extracted as single ion mode (SIM) from TIC and smoothened using Savitzky-Golay algorithms (**Fig. S3**).

ESI^{+ve} mass spectra extracted from standard run were extracted from retention time (12.13min) specific to vindoline and were stored in a library of LC solution and matched with sample run (**Fig. S4**). The similarity index quantitatively expressed the difference between spectrum of an unknown and a spectrum registered in library (Gupta et al., 2012, Srivastava et al., 2014). Following algorithm was used for calculating the similarity index between spectra-

$$SI = \left[1 - \frac{\sum_{m/z} \left| Iu(m/z) - (It(m/z) \right|}{\sum_{m/z} \left| It(m/z) + It(m/z) \right|} \right] \times 100$$

Where *Iu*(m/z): relative spectral intensity for mass (m/z) of the mass spectrum of an unknown sample; *It*(m/z): relative spectral intensity for mass (m/z) of the mass spectrum registered in a library.

If the patterns of the two mass spectra were identical, the similarity index (SI) was 100, and conversely, if each were completely different, the similarity index was 0. The similarity index for natural control (NC), endophyte-free control (C), CATDLF5 and CATDLF6 was found to be 66, 67, 74 and 76, respectively (**Fig. S4**), which further confirmed the specificity of vindoline quantification in addition to retention time and UV-spectra matching.

References

Gupta, S., Shanker, K. & Srivastava, S. K. HPTLC method for the simultaneous determination of four indole alkaloids in *Rauwolfia tetraphylla*: A study of organic/green solvent and continuous/pulse sonication. *J Pharmaceutical Biomed Anal* **66**, 33-39 (2012).

Srivastava, P., Ajayakumar, P. V. & Shanker K. Box-Behnken design for optimum extraction of biogenetic chemicals from *P. Lanceolata* with an Energy Audit (Thermal × Microwave × Acoustic): A case study of HPTLC determination with additional specificity using on-line/off-line coupling with DAD/NIR/ESI-MS. *Phytochemical Analysis* **25**, 551-560 (2014).

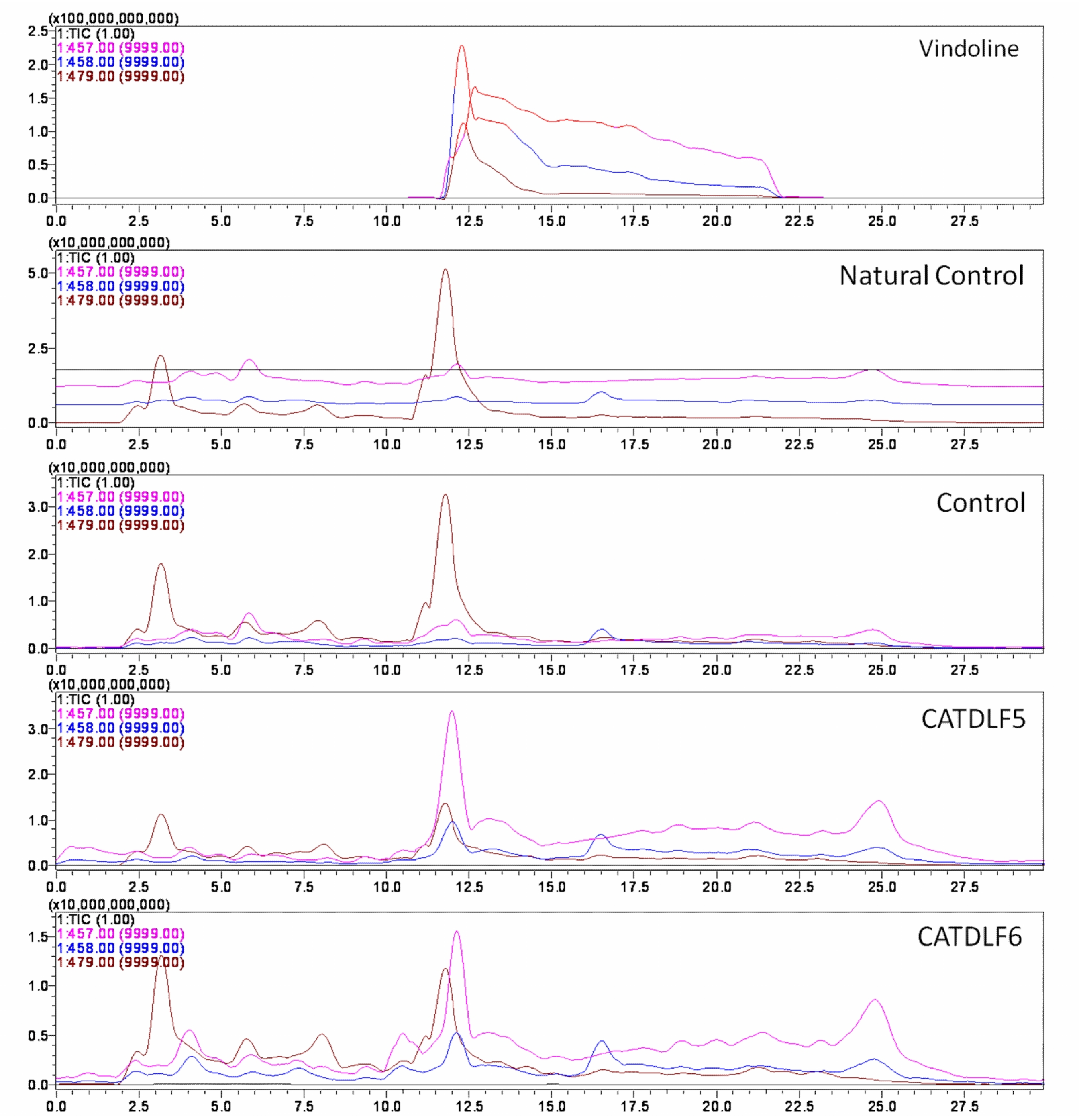

Supplementary Fig. S3: LC-MS chromatograms i.e. total ion chromatograms (TIC) and single ion mode (SIM) at 458 m/z $(M+H)^+$ of reference vindoline and samples confirming the specificity of vindoline determination.

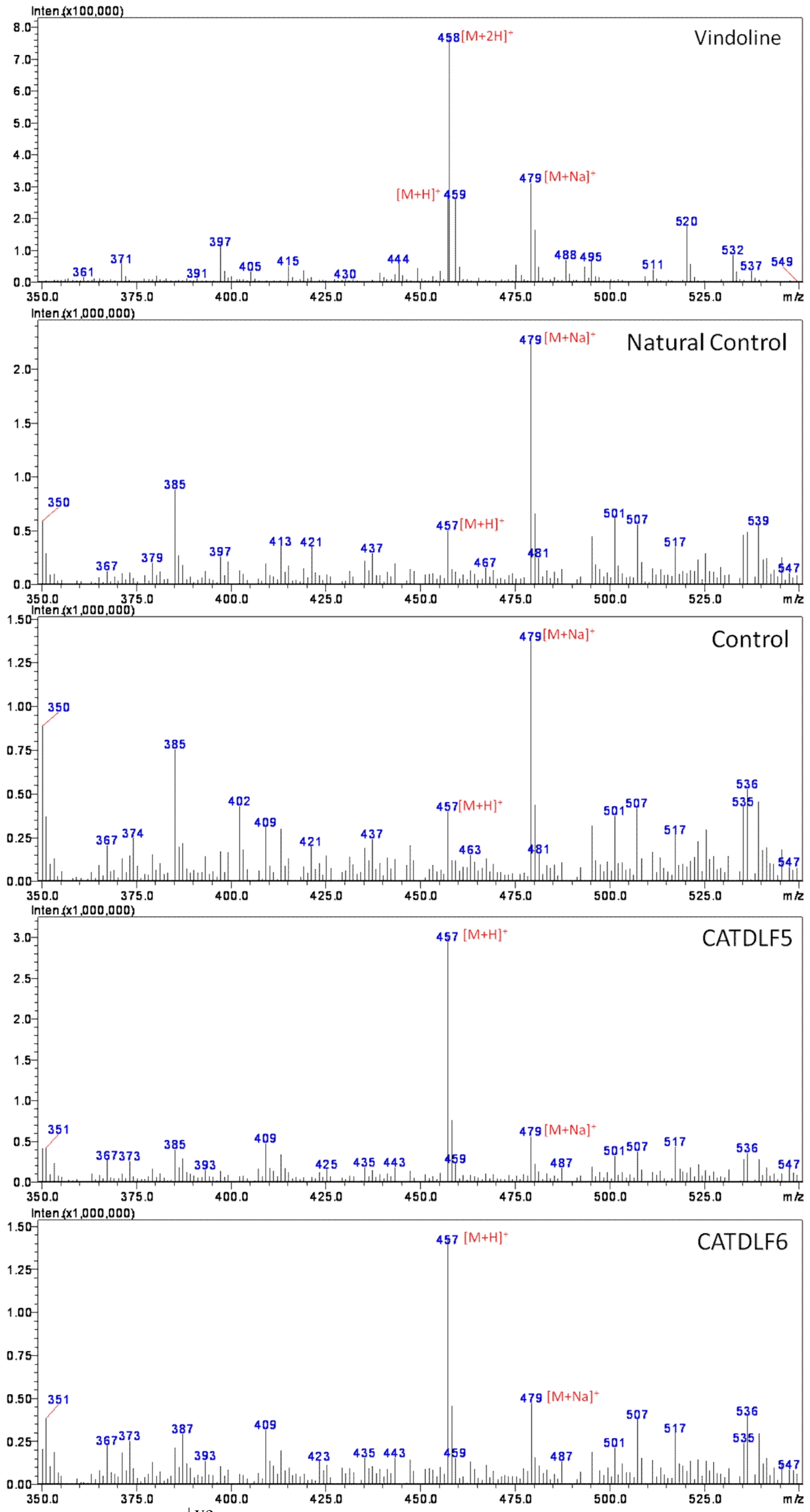

Supplementary Fig. S4: ESI^{+ve} mass spectra of reference vindoline and samples.
